# Antibody Binding and Neutralization of Live SARS-CoV-2 Variants Including BA.4/5 Following Booster Vaccination of Patients with B-cell Malignancies

**DOI:** 10.1158/2767-9764.CRC-22-0471

**Published:** 2022-12-22

**Authors:** Andres Chang, Akil Akhtar, Lilin Lai, Victor M. Orellana-Noia, Susanne L. Linderman, Ashley A. McCook-Veal, Jeffrey M. Switchenko, Manpreet Saini, Rajesh M. Valanparambil, Kristie A. Blum, Pamela B. Allen, Mary Jo Lechowicz, Jason T. Romancik, Amy Ayers, Alyssa Leal, Colin B. O'Leary, Michael C. Churnetski, Katelin Baird, Melissa Kives, Jens Wrammert, Ajay K. Nooka, Jean L. Koff, Madhav V. Dhodapkar, Mehul S. Suthar, Jonathon B. Cohen, Rafi Ahmed

**Affiliations:** 1 Department of Hematology and Medical Oncology, Winship Cancer Institute, Emory University School of Medicine, Atlanta, Georgia.; 2 Emory Vaccine Center, Department of Microbiology and Immunology, Emory University School of Medicine, Atlanta, Georgia.; 3 Department of Pediatrics, Emory University Schools of Medicine, Atlanta, Georgia.; 4 Emory National Primate Research Center, Atlanta, Georgia.; 5 Department of Biostatistics and Bioinformatics, Rollins School of Public Health, Emory University, Atlanta, Georgia.; 6 International Centre for Genetic Engineering and Biotechnology (ICGEB), New Delhi, India.; 7 Department of Lymphoma and Myeloma, The University of Texas MD Anderson Cancer Center, Houston, Texas.

## Abstract

Patients with non–Hodgkin lymphoma and chronic lymphocytic leukemia (NHL/CLL) elicit inadequate antibody responses after initial SARS-CoV-2 vaccination and remain at high risk of severe COVID-19 disease. We investigated IgG, IgA, and IgM responses after booster vaccination against recent SARS-CoV-2 variants including Omicron BA.5 in 67 patients. Patients had lower fold increase and total anti-spike binding titers after booster than healthy individuals. Antibody responses negatively correlated with recent anti-CD20 therapy and low B-cell numbers. Antibodies generated after booster demonstrated similar binding properties against SARS-CoV-2 variants compared with those generated by healthy controls with lower binding against Omicron variants. Importantly, 43% of patients showed anti-Omicron BA.1 neutralizing antibodies after booster and all these patients also had anti-Omicron BA.5 neutralizing antibodies. Patients with NHL/CLL demonstrated inferior antibody responses after booster vaccination, particularly against Omicron variants. Prioritization of prophylactic and treatment agents and vaccination of patients and close contacts with updated vaccine formulations are essential.

**Significance:**

Limited data exist on antibody responses against current SARS-CoV-2 variants after booster vaccination in patients with NHL/CLL. We showed inferior antibody responses against Omicron variants after booster vaccination in these patients but some generated anti-Omicron titers. This stresses the importance of vaccinating patients with updated formulations.

## Introduction

Patients with hematologic malignancies remain at increased risk of breakthrough infections, severe disease, and death from SARS-CoV-2 infections (1–7), particularly in those lacking detectable anti-SARS-CoV-2 antibodies after vaccination (8). We and others have reported that patients with lymphoid malignancies like B-cell non–Hodgkin lymphoma, chronic lymphocytic leukemia (NHL/CLL), and multiple myeloma have impaired antibody responses after the initial two-dose mRNA vaccination course, which are more pronounced when measured against SARS-CoV-2 variants including B.1.1.529 (Omicron; refs. 9–14). Patients with NHL/CLL and multiple myeloma are uniquely susceptible as they commonly receive therapies that either deplete B cells and plasma cells (e.g., anti-CD20 mAbs) or directly interfere with B-cell signaling pathways (15). Previous studies have reported reduced antibody responses after booster vaccination in patients with NHL/CLL, though these studies were limited largely to responses measured against the original strain or variants that are no longer in circulation (12, 13, 16–20). Fendler and colleagues recently reported poor neutralizing antibody responses against Omicron variant BA.1 after booster in a heterogeneous group of patients with hematologic malignancies when compared with solid tumor patients (18). However, most patients in that study were initially vaccinated with ChAdOx1 nCoV19, a vaccine with an inferior efficacy compared with mRNA-1273 (Moderna) and BNT162b2 (Pfizer/BioNTech; refs. 19, 21). In addition, analysis of antibody responses specifically in patients with NHL/CLL was limited and the effectiveness against more recent Omicron variants was not reported. Thus, antibody responses after SARS-CoV-2 booster vaccination in patients with NHL/CLL remain incompletely characterized. In this study, we sought to determine the effect of booster mRNA vaccination on IgG, IgA, and IgM antibody binding and live-virus neutralizing titers against SARS-CoV-2 in patients with NHL/CLL, with particular focus on recent Omicron variants. In addition, we also sought to determine the clinical characteristics in this population that may predict booster responses.

## Materials and Methods

### Patient Samples and Sample Processing

Information on the patient cohort has been published previously (9). Blood samples from patients with NHL/CLL at the Winship Cancer Institute (WCI) of Emory University after written informed consent under protocols approved by Emory University Institutional Review Board (IRB). Healthy volunteers’ samples were collected by Emory Children's Center in Atlanta after written informed consent also under approved protocols by Emory University IRB. Studies were conducted in accordance with the Declaration of Helsinki. Samples were collected, processed, and stored as described previously (9) and subsequent experiments were performed in a blinded fashion. Clinical data were obtained from the patient's electronic medical records.

### Viruses and Cells

VeroE6-TMPRSS2 cells were generated and cultured as described previously (22). nCoV/USA_WA1/2020 (WA/1), closely resembling the original Wuhan strain was propagated from an infectious SARS-CoV-2 clone as described previously (23). icSARS-CoV-2 was passaged once to generate a working stock. The BA.1 variant was isolated and propagated as described previously (22, 24). The BA.5 isolate was kindly provided by Dr. Richard Webby (St Jude Children's Research Hospital), plaque purified, and propagated once in VeroE6-TMPRSS2 cells to generate a working stock. All viruses used were deep sequenced and confirmed as described previously (22).

### Antibody Binding Assay

Antibody binding titters against SARS-CoV-2 spike, spike N-terminal domain (NTD), spike receptor binding domain (RBD), nucleocapsid, and the spike or RBD proteins of SARS-CoV-2 variants of concern were measured using a multiplex assay as described previously (9) and according to manufacturer's protocol (Mesoscale Discovery). Briefly, the plates were blocked with Blocker A in a shaker for at least 30 minutes and washed three times with wash buffer afterward. Plasma samples diluted at 1:2,000 or 1:10,000 were added to the plate and incubated shaking at room temperature for 2 hours. Plates were then incubated with 50 μL/well of Sulfo-Tag anti-human IgG, IgA, or IgM detection antibody (Mesoscale Discovery) at room temperature for 1 hour. The plates were read on the MSD instrument immediately after addition of 150 μL/well of MSD GOLD read buffer. Antibody binding titers are reported as arbitrary units/mL (AU/mL) based on normalization to a standard curve. Titer values above the fit curve range were manually set to the maximum titer provided by the curve. Background antibody binding levels were determined previously using measurements from 41 NHL/CLL patient samples obtained prior to February 1, 2020 (9).

### Focus Reduction Neutralization Test

Focus reduction neutralization test (FRNT) assays were performed as described previously (9, 22, 25, 26). Briefly, plasma samples were serially diluted and incubated with 100–200 Fluorescent Focus Unit (FFU) of WA1/2020, B.1.617.2, B.1.1.529, or B.1.1.529.BA.5 at 37°C for 1 hour. The virus-antibody mixture was then added to VeroE6-TMPRSS2 cells and incubated at 37˚C for 1 hour. The postincubation, virus-antibody mixture was removed and replaced by a 0.85% methylcellulose (Sigma-Aldrich, #M0512-250G) overlay. Cells were incubated at 37°C for 18 to 40 hours, after which the methylcellulose overlay was removed. The cells were washed, fixed with 2% paraformaldehyde, permeabilized, and incubated with Alexafluor-647–conjugated anti-SARS-CoV-2 spike antibody (CR3022-AF647, generated in house) for up to 4 hours at room temperature. Cells were then washed and visualized on an ELISPOT reader (CTL Analyzer). Antibody neutralization was quantified by counting the number of foci for each sample using Virodot. Neutralization titers were calculated as follows: 1 − (ratio of the mean number of foci in the presence of plasma and foci at the highest dilution of respective sample). Specimens were tested in duplicate. Samples that do not neutralize at the limit of detection at 50% are plotted at 10 and were used for geometric mean calculations.

### Flow Cytometry

Fresh whole blood was incubated with surface antibody cocktail containing the following: anti-CD19, anti-CD20, anti-CD3, anti-CD5, anti-CD8 (catalog nos. 302240, 302326, 300439, 300626, and 301048 from BioLegend, respectively) and anti-CD45 and anti-CD4 (catalog nos. 555482 and 562658 from BD Biosciences, respectively) in TruCount tubes (BD) and fixed with 1× FACS/Lyse (BD) as described previously (9). Samples were acquired on a LSR II (BD) or an Aurora (Cytek Biosciences) instrument and analyzed using FlowJo v10 (RRID:SCR_008520).

### Statistical Analysis

Statistical analysis was conducted using Graphpad Prism V9 (RRID:SCR_002798), and SAS 9.4 (RRID:SCR_008567). Significance level was set at *P* < 0.05, two tailed, for all analyses. Descriptive statistics were performed to tabulate patients’ demographic and clinical characteristics. Frequency and percentage, mean and SD or median with 95% confidence interval or interquartile range were reported on the basis of the data structure of variable. Statistical differences were assessed by group using one-way ANOVA, Brown–Forsythe and Welch ANOVA test, mixed-effect analysis with Geisser–Greenhouse correction, Friedman, Student *t* test, or Mann–Whitney test, when appropriate. Multiple comparisons are accounted for by utilizing Dunnet T3, Šidák, or Dunn test based on the selected statistical test and validity of assumptions. The strength of association between laboratory biomarkers was tested with Pearson or Spearman test, or nonlinear regression. For the univariate analysis, the outcome was defined as Spike IgG ≥500 AU/mL and ≥0.5 log_10_ increase after booster vaccination. Appropriate statistical tests were selected by validity of assumptions for the variables in analyses. The plots of the residuals (Q-Q plots) from each variable were used to examine to determine violations of assumptions and selection of appropriate statistical methods, for example, parametric or nonparametric statistical methods.

### Data Sharing Statement

Individual participant data that underlie the results reported in this article, after deidentification and study protocol will be made available beginning 3 months and ending 5 years following article publication to investigators whose proposed use of the data has been approved by an independent review committee (“learned intermediary”) identified for this purpose to achieve aims in the approved proposal. Proposals should be directed to the corresponding author; to gain access, data requestors will need to sign a data access agreement.

## Results

### Study Participants

A total of 67 patients with NHL/CLL (30 with B-cell NHL and 37 with CLL) were enrolled, 58% were male and 42% were female (Supplementary Table S1). Median age was 69 and most were White. Most patients initially received either the Moderna (61%) or the Pfizer/BioNTech (37%) vaccine, and 1 patient received Ad26.COV2.S (Janssen). A total of 90% of patients opted to receive a homologous booster vaccine. Median time between completion of initial vaccination series and booster vaccine was 195 days. In this cohort, 16% of patients had not received any lymphoma-directed therapy. Among patients with prior or current exposure to lymphoma-directed therapies, 64% received an anti-CD20 mAb (28% receiving treatment <1 year prior to initial vaccination), 55% had prior cytotoxic chemotherapy, 15% were receiving Bruton tyrosine kinase inhibitors (BTKi), and 13% of patients were receiving Bcl-2 inhibitors (Bcl-2i). Only 8 patients had prior cellular therapy (autologous or allogeneic stem cell transplantation, or chimeric antigen receptor T cell, CAR-T cell therapy). Importantly, none received passive immunization with anti-SARS-CoV-2 mAbs (such as sotrovimab or tixagevimab/cilgavimab) during the study.

### Binding Antibody Responses After Booster Vaccination

IgG, IgA, and IgM binding titers to the full-length spike, the RBD, and the NTD of the spike protein of the original SARS-CoV-2 B.1 strain were measured as described previously (9, 26). Anti-nucleocapsid antibodies were also measured to identify patients with prior SARS-CoV-2 infections. Six patients showed elevated anti-nucleocapsid IgG titers (Supplementary Fig. S1A). Minimal changes were observed in anti-nucleocapsid IgG titers before and after booster vaccination in the subset of patients with available longitudinal samples (Supplementary Fig. S1B).

**FIGURE 1:**
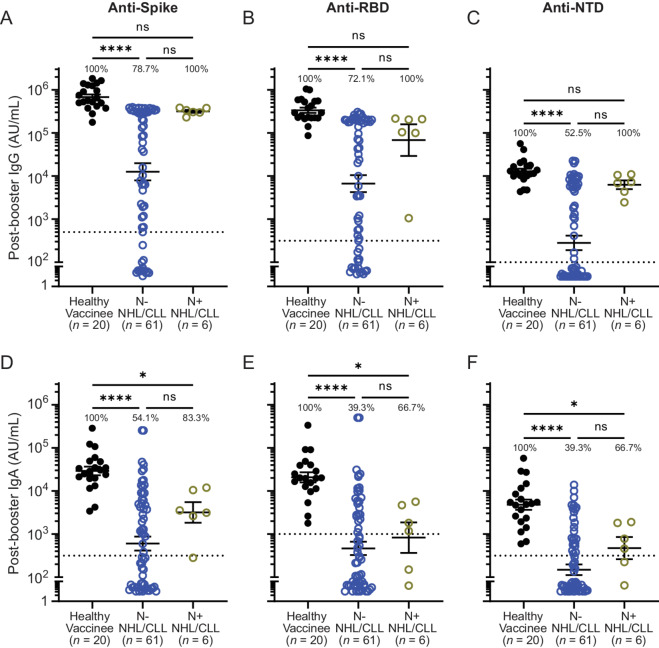
Lower antibody binding titers after booster vaccination are observed in patients with NHL/CLL compared with healthy controls. IgG (**A–C**), and IgA (**D–F**) binding titers against full-length spike protein (**A** and **D**) and its RBD (**B** and **E**) and NTD (**C** and **F**) after booster vaccination in patients based on presence or absence of evidence of prior infection (gold and blue, respectively). Percentages indicate percent of people showing titers above prepandemic levels (dotted line; ref. 9). Error bars = geometric mean ± 95% confidence interval (CI). *, *P* ≤ 0.05; **, *P* ≤ 0.01; ***, *P* ≤ 0.001; ****, *P* ≤ 0.0001 by using Brown–Forsythe and Welch ANOVA tests and using Dunnett T3 to correct for multiple comparisons or Kruskall–Wallis test and using Dunn to correct for multiple comparisons as appropriate. For all graphs: blue = nucleocapsid negative, gold = nucleocapsid positive, black = binding titers from healthy vaccinees without prior evidence of infection who also received mRNA booster vaccination (control). Horizontal dotted line = background antibody titers determined from prepandemic samples.

While healthy controls universally mounted robust IgG and IgA responses, patients with NHL/CLL exhibited a variable response, with a significant proportion mounting no discernible responses after booster vaccination (Fig. 1). IgG seropositivity rates (i.e., percent of individuals with titers above prepandemic controls) against spike, RBD, and NTD after booster were 78.7%, 72.1%, and 52.5%, respectively in anti–nucleocapsid-negative patients compared with 100% in healthy individuals (Fig. 1A–C). Geometric mean IgG binding titers against full-length spike, RBD, and NTD were reduced by 54-, 50-, and 46-fold, respectively in patients with NHL/CLL compared with healthy controls. IgA seropositivity rates ranged from 39.3% to 54.1% (Fig. 1D–F). Anti-spike, anti-RBD, and anti-NTD IgA binding titers were reduced 48-, 44-, and 32-fold, respectively. IgM titers were lower than IgG and IgA titers in healthy controls but were further decreased in patients with NHL/CLL by 3- to 4-fold (Supplementary Fig. S1C–S1E). Patients with evidence of prior infection (i.e., anti-nucleocapsid positive) showed higher seropositivity rates and appeared to have higher binding titers compared with anti–nucleocapsid-negative patients (Fig. 1A–F; Supplementary Fig. S1C–S1E) though numbers were small. These data indicate that patients with NHL/CLL exhibit lower antibody binding titers after booster vaccination compared with healthy controls.

**FIGURE 2:**
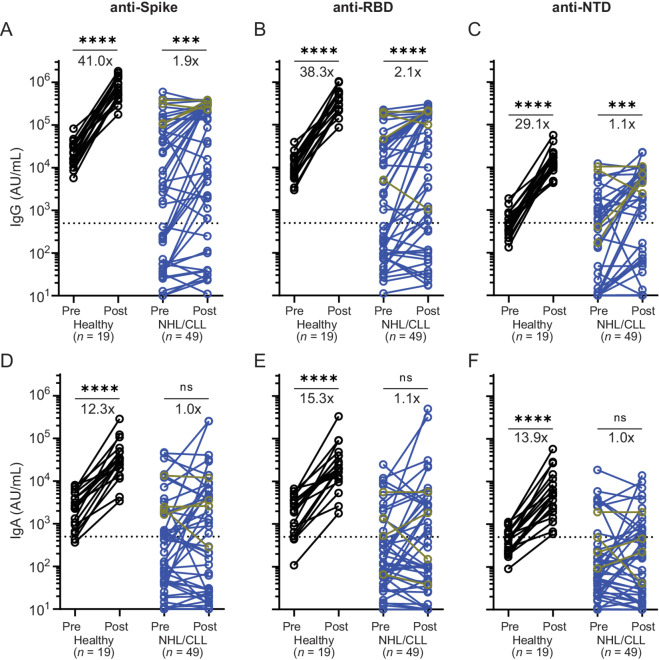
Diminished effect of booster vaccination on antibody titers in patients with NHL/CLL. IgG (**A–C**) and IgA (**D–F**) binding titers against full-length spike protein (**A** and **D**) and its RBD (**B** and **E**) and NTD (**C** and **F**) before and after booster vaccination in paired nucleocapsid positive (gold) and nucleocapsid-negative (blue) NHL/CLL patient samples (*n* = 49). Numbers represent median fold change from pre-booster titer after receipt of a booster vaccine. *, *P* ≤ 0.05; ***, *P* ≤ 0.001; ****, *P* ≤ 0.0001 by using mixed-effects analysis with Geisser–Greenhouse correction and using Šidák to correct for multiple comparisons. For all graphs: blue = nucleocapsid negative, gold = nucleocapsid positive, black = binding titers from healthy vaccinees without prior evidence of infection who also received mRNA booster vaccination (control). Horizontal dotted line = background antibody titers determined from prepandemic samples.

To better determine the effect of booster vaccination on antibody titers, paired analyses were conducted in the 49 patients for whom pre- and post-booster vaccination samples were available (Fig. 2). Anti-spike IgG titers increased by a median of 1.9-fold after booster vaccination in patients compared with 41-fold in healthy vaccinees (Fig. 2A). Significant differences were also observed in anti-RBD (2.1- vs. 38.3-fold) and anti-NTD (1.1- vs. 29.1-fold) IgG titers (Fig. 2B and C). Median IgA titers increased 12- to 15-fold in healthy controls but did not increase in patients with NHL/CLL (Fig. 2D–F). However, 18% and 14% of patients achieved IgG and IgA changes comparable with healthy controls, respectively. Minimal changes were seen in IgM binding titers for both groups (Supplementary Fig. S1F–S1H).

IgG binding titers against full-length spike closely correlated with titers against RBD and NTD (Supplementary Fig. S2A). Furthermore, anti-spike IgG also correlated with anti-spike IgA binding titers (*R*^2^ = 0.6222), though IgA titers were lower in nearly all patients (Supplementary Fig. S2B). Correlation between anti-spike IgG and IgM was less robust (*R*^2^ = 0.3709; Supplementary Fig. S2C). Overall, these data show that many patients with NHL/CLL mount only minimal or modest antibody responses to booster vaccination, on average achieving much lower fold change and post-boost titers than healthy controls.

### Longitudinal Antibody Titers After Booster Vaccination and Clinical Characteristics Associated with Response

Given that anti-spike IgG binding titers closely correlated with anti-RBD and anti-NTD IgG, and with IgA titers (Supplementary Fig. S2), subsequent analyses were performed using anti-spike IgG. Among the 49 patients with available longitudinal samples, 13 demonstrated high titers (≥100,000 AU/mL) before booster (Supplementary Fig. S3A). These patients did not attain substantial increases in antibody titers after booster vaccination. Eleven patients in this group had either not yet received any lymphoma-directed therapy (*n* = 5) or received anti–CD20-directed therapy >1 year prior to vaccination (*n* = 6; Supplementary Table S2). Two patients received anti–CD20-directed therapy within 1 year, but both had prior SARS-CoV-2 infection. Indeed, all anti–nucleocapsid-positive patients showed high titers prior to booster vaccination and were included in this group. Of the remaining patients, we observed that titers increased by at least 3.16-fold (i.e., 0.5 log_10_) after booster in 18 patients while the other 18 did not experience this response (Supplementary Fig. S3B).

To determine the clinical factors that predict vaccine response, a series of univariable analyses were performed. No significant differences were observed on the basis of patient's age, gender, race, or lymphoma histology (Supplementary Table S3). Ongoing use of BTKi or Bcl-2i, or prior cellular therapies did not correlate with vaccine response, though sample size was small. Among the factors evaluated, only receipt of prior anti-CD20 mAb ≤1 year of initial vaccination strongly correlated with antibody responses after booster vaccination. Indeed, we found that circulating conventional B-cell numbers prior to booster vaccination correlated with anti-spike IgG titers after vaccination (Supplementary Fig. S4), as anti-CD20 mAbs deplete circulating malignant and nonmalignant B cells. No correlation was observed between antibody response and timing between completion of initial vaccination series and receipt of booster vaccine. Together, these results demonstrate that patients with high baseline antibody titers are unlikely to experience a substantial increase in titers after booster vaccination, and that antibody responses correlate with conventional B-cell numbers in the blood, which are affected by exposure to anti-CD20 mAbs.

### Antibody Binding Against Variant SARS-CoV-2 Spike Proteins

**FIGURE 3:**
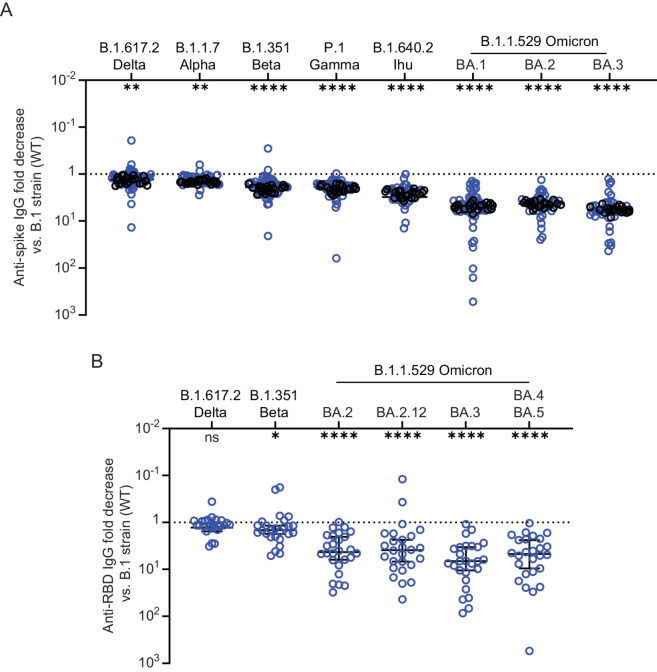
Reductions in antibody binding titers against spike protein of SARS-CoV-2 variants is similar between NHL/CLL and healthy controls. **A,** Fold decrease in antibody titers from WT B1. Strain (Wuhan, WT) against SARS-CoV-2 variants is similar between patients with NHL/CLL (*n* = 45 or 31) and healthy controls (*n* = 20) but greater variability is observed in patients with NHL/CLL. **B,** Fold decrease in binding antibody titers from WT RBD against RBD from variant SARS-CoV-2 including BA.5, which has identical spike protein as BA.4 (ref. 27; *n* = 26). Error bars = median ± 95% CI. *, *P* ≤ 0.05; **, *P* ≤ 0.01; ****, *P* ≤ 0.0001 versus WT by using Kruskal–Wallis test and using Dunn to correct for multiple comparisons.

Given that the predominant circulating SARS-CoV-2 variant has significantly changed over the course of the pandemic, we analyzed post-booster antibody binding capacity to spike proteins of more recent SARS-CoV-2 variants including B.1.617.2 AY.4.2 (Delta) and the Omicron variants BA.1, BA.2, and BA.3. Consistent with our findings in the ancestral B.1 strain (wildtype, WT), post-booster binding titers against SARS-CoV-2 variants were also lower and more variable in patients with NHL/CLL than in healthy controls (Supplementary Fig. S5A). Geometric mean titers against the variant spike was reduced compared with WT spike, with most significant reductions observed in binding titers against spike from Omicron variants. However, similar fold reductions were observed in patients with NHL/CLL and healthy vaccinees (Fig. 3A). Binding titers against BA.1 was reduced by a median of 4.8-fold compared with WT in both patients with NHL/CLL and healthy controls. Titers against BA.1, BA.2, and BA.3 strains were largely equivalent. We also assessed the antibody binding capacity against the RBD of newer Omicron variants including BA.4 and BA.5, which share the same spike protein sequence (27). Titers against the RBD of these variants were significantly reduced compared with WT strain (Supplementary Fig. S5B). Greater variability was observed in titers against Omicron strains, but the median fold reduction compared with the WT strain was similar (Fig. 3B). Overall, these results indicate that the antibodies generated by SARS-CoV-2 vaccines in patients with NHL/CLL have lower binding capacity against the spike proteins of SARS-CoV-2 variants.

### Live Virus Neutralization Against SARS-CoV-2 Variants

**FIGURE 4:**
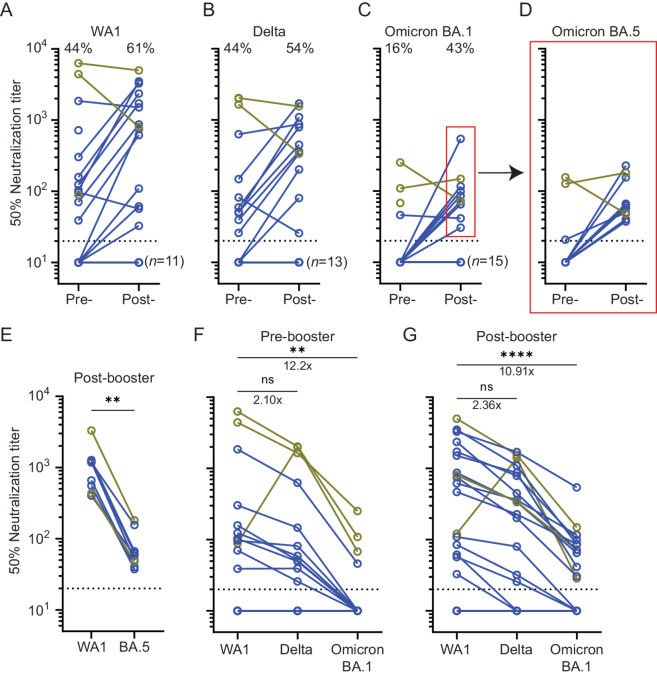
Minimal induction of live-virus neutralization titers against Omicron variants after booster vaccination. Neutralization titers against ancestral strain WA1 (**A**) improved after booster vaccination but less so against Delta (**B**), and Omicron BA.1 (**C**). However, more patients developed detectable neutralizing antibodies against all tested variants after booster vaccination. Red box = samples tested and depicted in **D**. Percentages = percent of patients with NHL/CLL with detectable titers. *n* = 28. **D,** Neutralization titers against Omicron BA.5 before and after booster vaccination among patients with detectable titers against Omicron BA.1 (*n* = 10). **E,** Titers against Omicron BA.5 were lower compared with WA1 in patients with detectable titers against Omicron BA.1 after booster vaccination. **, *P* ≤ 0.01, respectively using Wilcoxon test. Neutralization titers before (**F**) and after (**G**) booster vaccination against WA1, Delta, and Omicron variants in patients with NHL/CLL. Titers against Omicron remain significantly lower compared with WA1. However, the fold decrease in neutralizing antibody titers did not change after booster vaccination. **, *P* ≤ 0.01; ****, *P* ≤ 0.0001 by using Friedman test and using Dunn to correct for multiple comparisons. Number = fold change. For all graphs: Horizontal dotted line = limit of detection. Blue = nucleocapsid-negative patients. Gold = nucleocapsid-positive patients.

To determine the post-booster antibodies’ functional capacity to neutralize SARS-CoV-2 infection, we performed live-virus FRNT against the ancestral WA1/2020 strain (WA1) and the Delta, Omicron BA.1, and BA.5 variants in a subset of samples. The proportion of patients demonstrating neutralizing titers against WA1 increased from 44% to 61% after booster vaccination (Fig. 4A). Median titers increased 11-fold for WA1 and 8-fold against Delta in patients with detectable neutralizing antibodies (Fig. 4A and B). Notably, only 4 patients had detectable neutralization titers against BA.1 prior to booster vaccination (16%), three of which were anti-nucleocapsid positive. Neutralizing antibodies against BA.1 were detected in 43% of patients after booster vaccination (Fig. 4C). Importantly, patients with detectable titers against BA.1 after booster vaccination also had detectable neutralization titers against BA.5 (Fig. 3D), though titers against BA.5 were 15-fold lower than WA1 (Fig. 4E). Indeed, consistent with the binding data, neutralization titers were highest against WA1 and lowest against BA.1 before and after booster vaccination (Fig. 4F and G). The only exception to this trend was observed in a patient who was infected at a time where the Delta variant was predominant. This patient had higher neutralizing antibody titers against the Delta variant. Overall, the fold decrease in titers against variants did not change significantly and median titers against BA.1 before and after booster vaccine were 11- to 12-fold lower than WA1. Our data showed that many patients with NHL/CLL had no detectable neutralizing antibody titers against Omicron variants before booster vaccination but 43% of patients developed low Omicron neutralizing titers afterward.

## Discussion

We previously showed that patients with NHL/CLL have impaired antibody responses against SARS-CoV-2 and some variants of concern after two doses of mRNA vaccines (9). In this study, we demonstrated that antibody responses after booster vaccination are modest in this patient population, particularly against Omicron variants. Most patients had lower IgG, IgA, and IgM titers compared with healthy individuals and many showed no detectable antibody responses after booster vaccination. These findings emphasize that patients with NHL/CLL remain a vulnerable group in need for additional protection against SARS-CoV-2 infection even after getting a booster vaccine.

Our data showed that some patients with NHL/CLL had high antibody titers prior to booster and that these patients did not increase their titers after vaccination. This is likely due to antigen masking and subsequent clearance by the high amount of circulating antibodies, limiting exposure of spike-specific B cells to antigen and their ensuing response, as has been observed following influenza vaccination (28). In addition, we showed that anti-CD20 therapy within 1 year of vaccination and low circulating conventional B-cell numbers negatively correlated with antibody responses, indicating that these factors are also relevant for booster vaccines as it is for initial vaccination (9). These data emphasize that treatment history and circulating B-cell count are critical indicators that identify patients at the highest risk of having poor antibody responses to vaccines and increased susceptibility to severe infection. This has profound clinical implications for the care of patients with NHL/CLL or other conditions requiring immunosuppressive therapies such as anti-CD20 mAbs.

Previous reports have shown that neutralization titers against Omicron elicited by booster vaccines are lower compared with prior SARS-CoV-2 variants in the general population (29–31). Furthermore, most mAbs developed early in the COVID-19 pandemic have poor neutralizing activity against Omicron variants, including those approved for use in the prevention and treatment of SARS-CoV-2 infections (32). This underscores the ability of Omicron variants to evade antibodies produced against spike proteins from earlier strains and provides compelling rationale for using updated booster vaccines that target Omicron epitopes. The relationship between antibody titers and specificity and infection risk and/or disease severity still is still unclear but seronegative patients are at greater risk of breakthrough infections (8). The observation that a subset of patients with NHL/CLL do not respond to booster vaccines suggest that some patients, including those on anti–CD20-directed therapy, will likely not respond to an updated formulation, stressing the importance of vaccinating close contacts. In addition, development of broadly neutralizing antibodies for passive immunization is urgently needed to protect this high-risk population.

Our study has some limitations. First, this is a single-center study with a limited sample size of predominantly elderly, White individuals with a diagnosis of CLL or diffuse large B-cell lymphoma who have not undergone cellular therapies, limiting its generalizability. Second, most of our patients received homologous booster vaccines and thus we did not have power to assess antibody responses in heterologous vaccination. Third, our healthy vaccinee cohort is not age and race matched. It is therefore possible that the differences between healthy aged-matched individuals and those in our cohort are less significant than what we observed in this study. However, many patients in this study were not actively receiving lymphoma-directed therapies, suggesting some enrichment of patients with more indolent lymphomas or patients in remission. Patients whose lymphomas require treatment would be expected to exhibit lower responses than what is reported in this study. Finally, this study did not evaluate the effect of booster vaccinations on T-cell immunity. These studies are currently ongoing.

Overall, we showed that most patients with NHL/CLL have impaired antibody responses after SARS-CoV-2 booster vaccination compared with healthy vaccinees. The responses observed against Omicron variants were particularly poor but the use of the recently approved bivalent SARS-CoV-2 vaccines, which include mRNA sequences of the original and the BA.4/BA.5 spike proteins, may improve antibody responses against these variants in patients who are able to mount an antibody response. Measuring the antibody response to these vaccines in patients with NHL/CLL particularly against Omicron variants would be of great interest as these vaccines should generate recall responses from cross-reactive B cells and may also generate *de novo* responses. Patients who did not respond to previous vaccinations but are now recovering their B-cell repertoire may now have a positive antibody response. Thus, vaccination of patients and close contacts should remain a high priority. Development of passive immunization strategies using neutralizing antibodies against conserved regions of the spike protein is urgently needed to protect those who are unlikely to respond to vaccination.

## Supplementary Material

Supplementary Table ST1 Supplemental Table 1. Clinical and vaccination characteristics of NHL/CLL patients in this study

Supplementary Table ST2 Supplemental Table 2. Clinical characteristics of NHL/CLL patients with high anti-spike IgG titers prior to booster vaccination

Supplementary Table ST3 Supplementary Table 3. Univariate analyses of clinical variables associated with booster response

Supplementary Figure SF1 Supplemental Figure 1. Anti-nucleocapsid IgG and spike IgM titers in NHL/CLL patients.

Supplementary Figure SF2 Supplemental Figure 2. Positive correlations in antibody binding titers between anti-Spike IgG and anti-RBD, anti-NTD IgG, and anti-spike IgA and IgM

Supplementary Figure SF3 Supplemental Figure 3. Kinetics of serologic response after booster vaccination.

Supplementary Figure SF4 Supplemental Figure 4. Anti-spike binding IgG titers after booster correlate with conventional B cell numbers in the blood prior booster.

Supplementary Figure SF5 Supplemental Figure 5. Reduced IgG binding titers against the spike protein of SARS-CoV-2 variants in NHL/CLL patients.
