# Supplementary material for: Antibody Binding and Neutralization of Live SARS-CoV-2 Variants Including BA.4/5 Following Booster Vaccination of Patients with B-cell Malignancies
Source: Cancer Res Commun. 2022 Dec 22;2(12):1684–92. doi: 10.1158/2767-9764.CRC-22-0471 (PMC9833496; doi:10.1158/2767-9764.CRC-22-0471)
Supplement: Supplementary Table ST1 — Supplemental Table 1. Clinical and vaccination characteristics of NHL/CLL patients in this study [file crc-22-0471-s01.pdf]

Supplemental Table 1. Clinical and vaccination characteristics of NHL/CLL patients in this study

**Sex**	**n**	**%**
Female	28	41.8
Male	39	58.2
**Age (yrs)**		
Median (IQR)	69	59-75
Range	37-92	
**Race**		
White	55	82.1
Non-White	10	14.9
Unknown	2	3
**Lymphoma subtype**		
CLL/SLL	37	55.2
NHL (all subtypes)	30	44.8
DLBCL	12	17.9
FL	7	10.5
MCL	4	6
Other*	7	10.5
**Vaccine manufacturer - initial series**		
Pfizer	25	37.3
Moderna	41	61.2
Janssen	1	1.5
**Vaccine manufacturer - booster**		
Pfizer	23	37.3
Moderna	44	65.7
**Booster vaccine**		
Homologous	60	89.6
Heterologous	7	10.4
**Time between completion of initial vaccination series and booster (days)**		
Median (IQR)	195	(177-244)
Range	90-342	
**Any lymphoma-directed therapy**		
No	11	16.4
Yes	56	83.6
**Prior anti-CD20 monoclonal antibody**		
No	24	35.8
Yes	43	64.2
Yes, within 1 year pre-booster	19	28.4
Yes, >1 year pre-booster	24	35.8
**Prior cytotoxic chemotherapy**		
No	30	44.8
Yes	37	55.2
**Prior cellular therapy**		
No	59	88.1
Yes	8	11.9
Autologous SCT	5	7.5
Allogeneic SCT	2	3
CD19 CAR-T	2	3
**Ongoing Bcl-2 inhibitor therapy**		
No	58	86.6
Yes	9	13.4
**Ongoing BTK inhibitor therapy**		
No	57	85.1
Yes	10	14.9

**Includes (N): WM (3), MZL (1), BL (1), PTCL (1), CLL/DLBCL (1)*
