# Supplementary material for: Antibody Binding and Neutralization of Live SARS-CoV-2 Variants Including BA.4/5 Following Booster Vaccination of Patients with B-cell Malignancies
Source: Cancer Res Commun. 2022 Dec 22;2(12):1684–92. doi: 10.1158/2767-9764.CRC-22-0471 (PMC9833496; doi:10.1158/2767-9764.CRC-22-0471)
Supplement: Supplementary Table ST2 — Supplemental Table 2. Clinical characteristics of NHL/CLL patients with high anti-spike IgG titers prior to booster vaccination [file crc-22-0471-s02.pdf]

Supplemental Table 2. Clinical characteristics of NHL/CLL patients with high anti-spike IgG titers prior to booster vaccination

	n	%
**Gender**		
Female	6	46.2
Male	7	53.9
**Age -** median 67 (IQR 52-70)		
<65	6	46.2
≥65	7	53.9
**Race**		
White	11	84.6
Non-White	2	15.4
**Lymphoma subtype**		
CLL/SLL	10	76.9
NHL (all subtypes)	3	23.1
**Vaccine manufacturer - initial series**		
Pfizer/BioNTech	4	30.8
Moderna	9	69.2
**Vaccine manufacturer - booster**		
Pfizer/BioNTech	5	38.5
Moderna	8	61.5
**Booster Vaccine**		
Homologous	12	92.3
Heterologous	1	7.7
**Prior anti-CD20 monoclonal antibody**		
No	5	38.5
Yes	8	61.5
Yes, within 1 year pre-booster	2	25
Yes, >1 year pre-booster	6	75
**Prior cytotoxic chemotherapy**		
No	7	53.8
Yes	6	46.2
**Prior cellular therapy**		
No	13	100
Yes	0	0
**Ongoing Bcl-2 inhibitor therapy**		
No	13	100
Yes	0	0
**Ongoing BTK inhibitor therapy**		
No	11	84.6
Yes	2	15.4

High titers defined as anti-spike IgG ≥100,000 prior to booster vaccination.
