# Supplementary material for: Antibody Binding and Neutralization of Live SARS-CoV-2 Variants Including BA.4/5 Following Booster Vaccination of Patients with B-cell Malignancies
Source: Cancer Res Commun. 2022 Dec 22;2(12):1684–92. doi: 10.1158/2767-9764.CRC-22-0471 (PMC9833496; doi:10.1158/2767-9764.CRC-22-0471)
Supplement: Supplementary Table ST3 — Supplementary Table 3. Univariate analyses of clinical variables associated with booster response [file crc-22-0471-s03.pdf]

**Supplementary Table 3.** Univariate analyses of clinical variables associated with booster response

| | Responder (n)* / Evaluable (n)** | % Responder | OR | 95% CI | P |
|---|---|---|---|---|---|
| **Gender** | | | | | |
| Female | 9 / 17 | 52.9 | ref | - | - |
| Male | 9 / 19 | 47.4 | 1.25 | 0.34-4.64 | 0.74 |
| **Age (yrs)** | | | 1.04 | 0.97-1.11 | 0.25 |
| < 65 | 5 / 13 | 38.5 | ref | - | - |
| ≥65 | 13 / 23 | 56.5 | 2.08 | 0.52-8.34 | 0.3 |
| **Race** | | | | | |
| White | 14 / 27 | 51.9 | ref | - | - |
| Non-White | 3 / 7 | 42.9 | 0.7 | 0.13-3.72 | 0.67 |
| **Lymphoma subtype** | | | | | |
| CLL/SLL | 12 / 22 | 54.5 | ref | - | - |
| NHL (all subtypes) | 6 / 14 | 42.9 | 0.63 | 0.16-2.41 | 0.5 |
| **Vaccine manufacturer - initial series** | | | | | |
| Pfizer/BioNTech | 4 / 12 | 33.3 | ref | - | - |
| Moderna | 14 / 23 | 60.9 | 3.11 | 0.72-13.44 | 0.96 |
| Janssen | 0 / 1 | 0 | - | - | - |
| **Vaccine manufacturer - booster** | | | | | |
| Pfizer/BioNTech | 4 / 11 | 36.4 | ref | - | - |
| Moderna | 14 / 25 | 56 | 2.23 | 0.52-9.59 | 0.28 |
| **Booster vaccine** | | | | | |
| Homologous | 18 / 34 | 52.9 | - | - | - |
| Heterologous | 0 / 2 | 0 | - | - | - |
| **Prior anti-CD20 monoclonal antibody** | | | | | |
| No | 6 / 9 | 66.7 | ref | - | - |
| Yes | 12 / 27 | 44.4 | 0.4 | 0.08-1.94 | 0.26 |
| Yes, within 1 year pre-booster | 3 / 14 | 21.4 | ref | - | - |
| Yes, >1 year pre-booster | 9 / 13 | 69.2 | 8.25 | 1.45-46.86 | **0.02** |
| **Prior cytotoxic chemotherapy** | | | | | |
| No | 8 / 15 | 53.3 | ref | - | - |
| Yes | 10 / 21 | 47.6 | 0.8 | 0.21-3.00 | 0.74 |
| **Prior cellular therapy** | | | | | |
| No | 15 / 30 | 50 | ref | - | - |
| Yes | 3 / 6 | 50 | 1 | 0.17-5.77 | 1 |
| **Ongoing Bcl-2 inhibitor therapy** | | | | | |
| No | 16 / 29 | 55.2 | ref | - | |
| Yes | 2 / 7 | 28.6 | 0.33 | 0.05-1.96 | 0.22 |
| **Ongoing BTK inhibitor therapy** | | | | | |
| No | 17 / 32 | 53.1 | ref | - | - |
| Yes | 1 / 4 | 25 | 0.29 | 0.03-3.14 | 0.31 |

** Responder defined as anti-spike IgG ≥ 500 AU/mL and a 0.5 log10 increase in antibody titers at least 3 days after booster vaccination*

***Response evaluability defined as at least one sample pre- and post-booster. Analysis excludes patients in the "high-titer" group*
