# Supplementary material for: Antibody Binding and Neutralization of Live SARS-CoV-2 Variants Including BA.4/5 Following Booster Vaccination of Patients with B-cell Malignancies
Source: Cancer Res Commun. 2022 Dec 22;2(12):1684–92. doi: 10.1158/2767-9764.CRC-22-0471 (PMC9833496; doi:10.1158/2767-9764.CRC-22-0471)
Supplement: Supplementary Figure SF1 — Supplemental Figure 1. Anti-nucleocapsid IgG and spike IgM titers in NHL/CLL patients. [file crc-22-0471-s04.pdf]

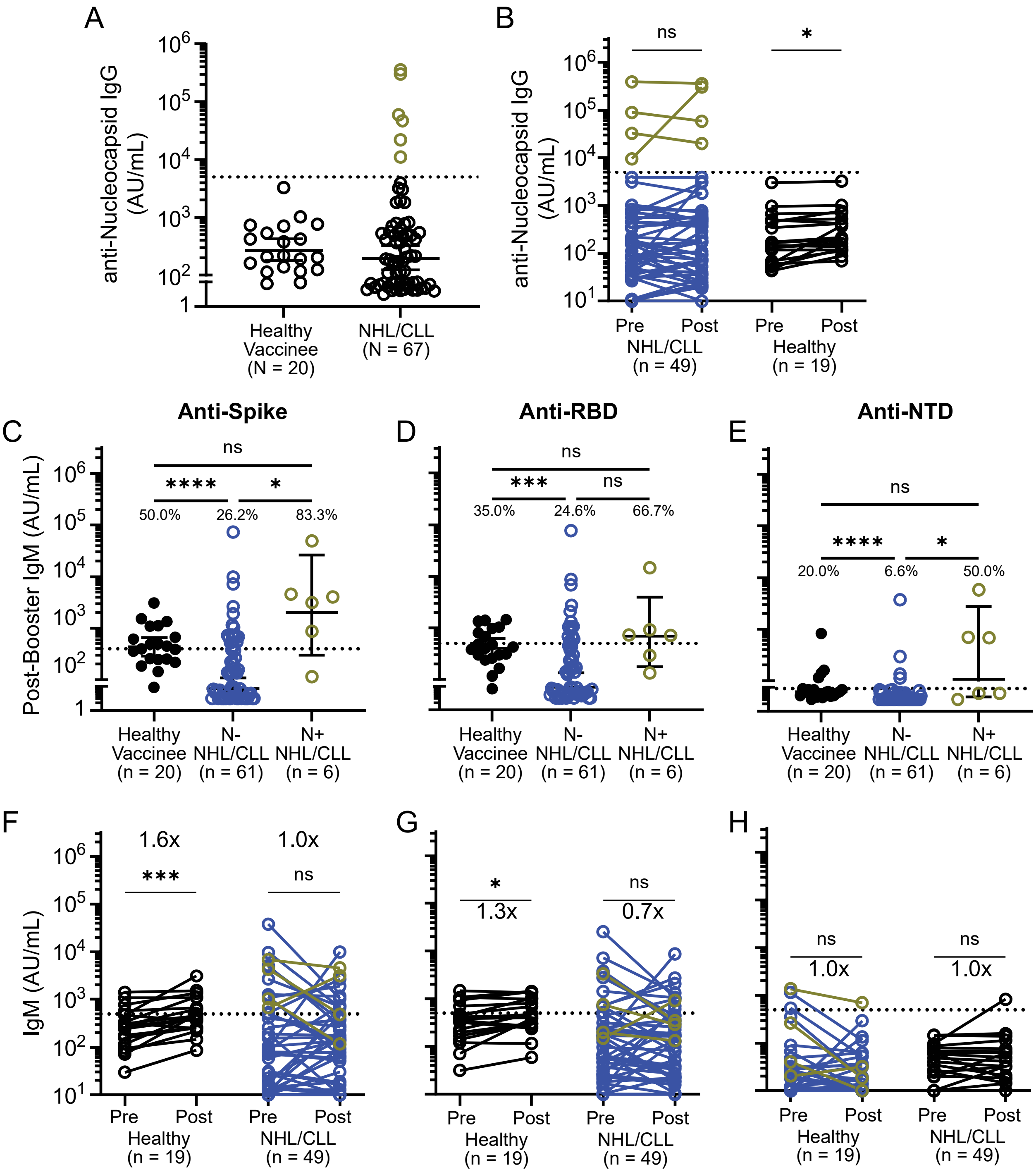

Supplemental Figure 1. Anti-nucleocapsid IgG and spike IgM titers in NHL/CLL patients. A) Anti-nucleocapsid IgG binding titers among NHL/CLL patients in this cohort and healthy controls. Gold = nucleocapsid positive patients, indicative of prior SARS-CoV-2 infection. B) Anti-nucleocapsid antibody titers among NHL/CLL patients and healthy controls at timepoints before and after booster vaccination. C-E) Binding titers against full-length spike protein (C) and its RBD (D) and NTD (E) after booster vaccination

in patients based on presence or absence of evidence of prior infection (gold and blue, respectively). Percentages indicate % of people showing titers above pre-pandemic levels (dotted line)[8]. F-H) IgM binding titers against spike (D), RBD (F), and NTD (G) before and after booster vaccination in paired nucleocapsid-positive (gold) and nucleocapsid-negative (blue) NHL/CLL patient samples (n = 49). Numbers represent median fold change from pre-booster titer after receipt of a booster vaccine. In all graphs: horizontal dotted line = background antibody titers determined from pre-pandemic samples. Error bars = geometric mean ± SEM. * = p≤0.05, ** = p≤0.01, *** = p≤0.001, **** = p≤0.0001 by using Brown-Forsythe and Welch ANOVA tests and using Dunnett T3 to correct for multiple comparisons or Kruskall-Wallis test and using Dunn's to correct for multiple comparisons as appropriate.
