# Supplementary material for: Antibody Binding and Neutralization of Live SARS-CoV-2 Variants Including BA.4/5 Following Booster Vaccination of Patients with B-cell Malignancies
Source: Cancer Res Commun. 2022 Dec 22;2(12):1684–92. doi: 10.1158/2767-9764.CRC-22-0471 (PMC9833496; doi:10.1158/2767-9764.CRC-22-0471)
Supplement: Supplementary Figure SF2 — Supplemental Figure 2. Positive correlations in antibody binding titers between anti-Spike IgG and anti-RBD, anti-NTD IgG, and anti-spike IgA and IgM [file crc-22-0471-s05.pdf]

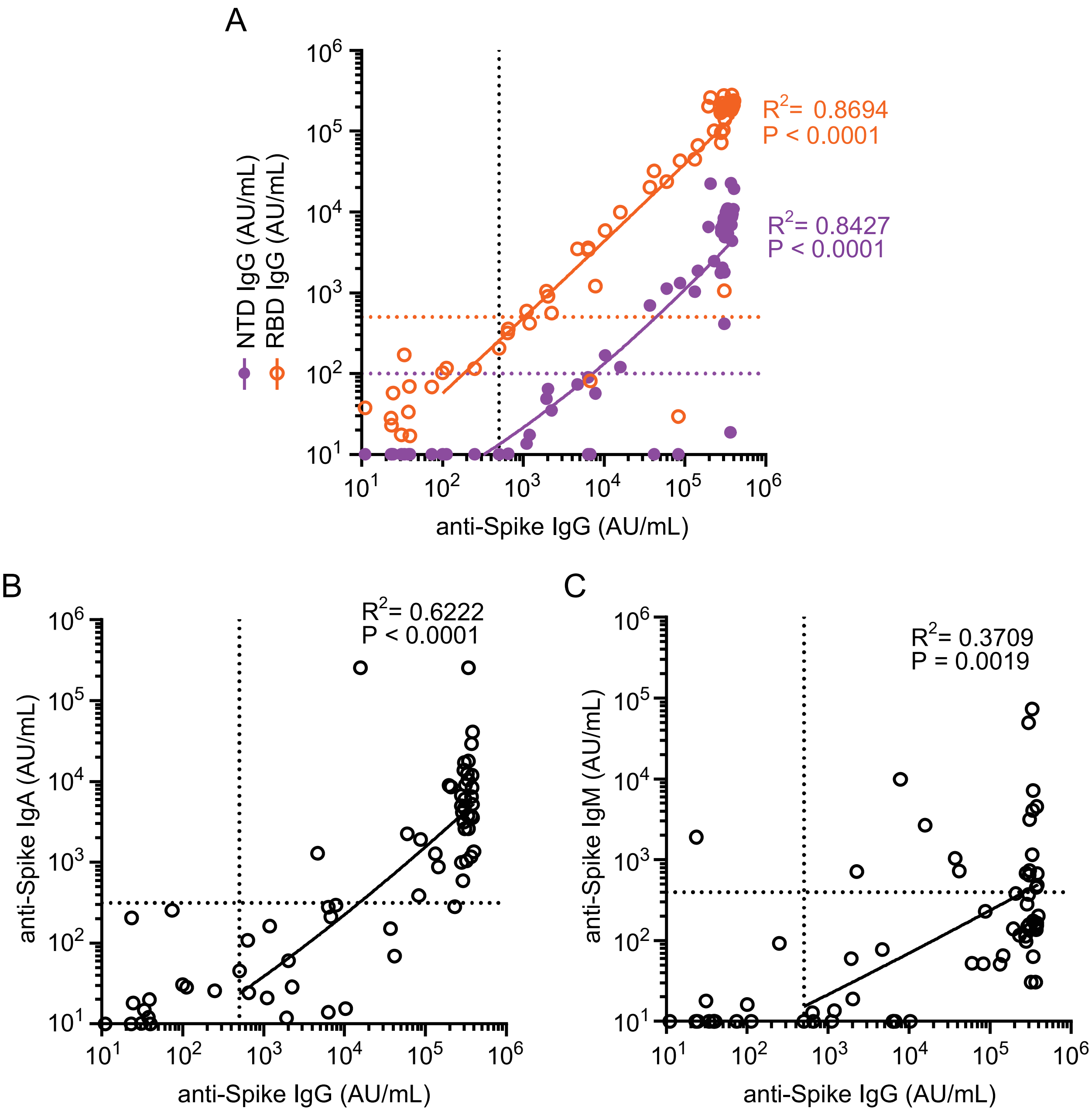

Supplemental Figure 2. Positive correlations in antibody binding titers between anti-Spike IgG and anti-RBD, anti-NTD IgG, and anti-spike IgA and IgM. A) IgG binding titers against full-length spike protein correlate with anti-RBD (orange) and anti-NTD (purple) binding IgG titers. B-C) Anti-spike IgG titers correlate better with anti-spike IgA (B) than IgM (C). Horizontal and vertical dotted lines = background antibody levels determined from pre-pandemic samples. Correlations were statistically significant using extra-sum-of-squares F test.
