# Supplementary material for: Antibody Binding and Neutralization of Live SARS-CoV-2 Variants Including BA.4/5 Following Booster Vaccination of Patients with B-cell Malignancies
Source: Cancer Res Commun. 2022 Dec 22;2(12):1684–92. doi: 10.1158/2767-9764.CRC-22-0471 (PMC9833496; doi:10.1158/2767-9764.CRC-22-0471)
Supplement: Supplementary Figure SF3 — Supplemental Figure 3. Kinetics of serologic response after booster vaccination. [file crc-22-0471-s06.pdf]

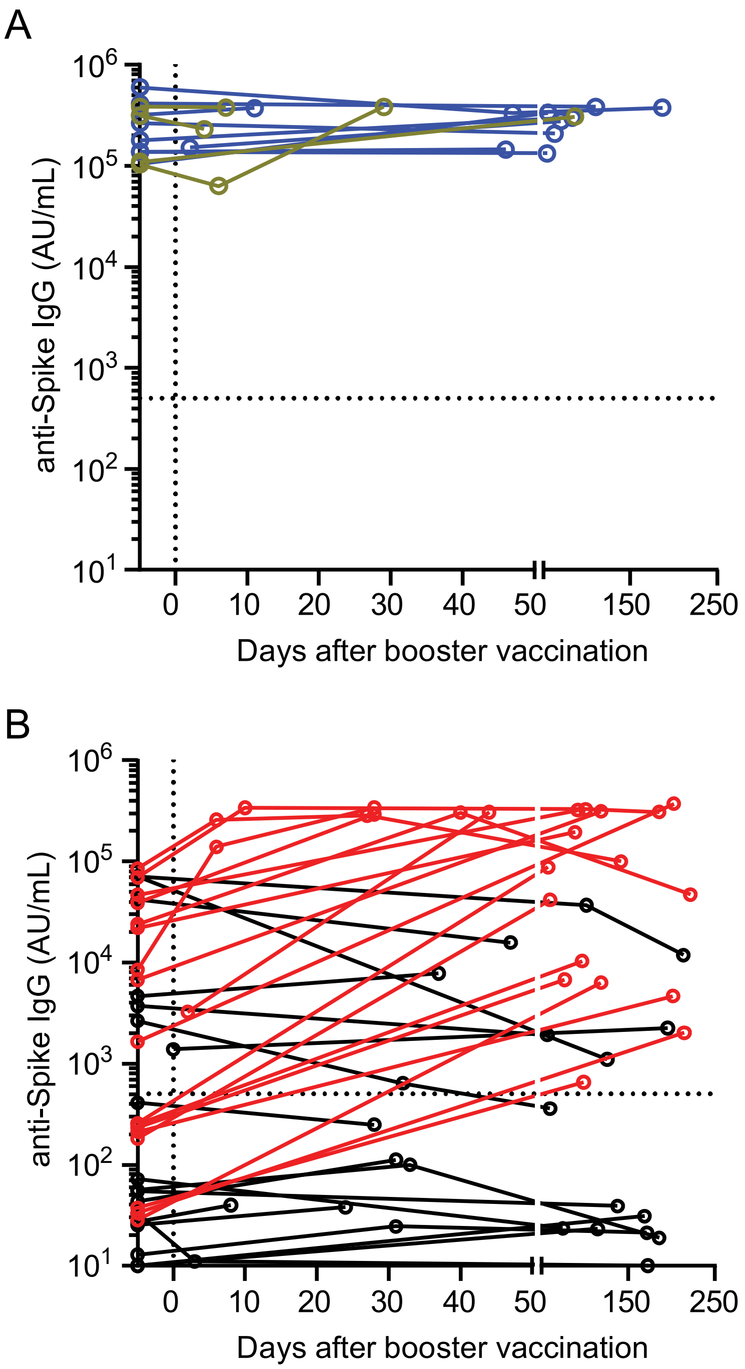

Supplemental Figure 3. Kinetics of serologic response after booster vaccination. A) Antibody titers over time of patients who had anti-spike IgG binding titers >100,000 AU/mL prior to booster vaccination. B) Longitudinal antibody response in NHL/CLL patients with pre-boost titers <100,000 AU/mL. Some patients experienced a >3.16-fold titer increase after booster vaccination (red) while others did not (black). Horizontal dotted line = background antibody levels determined from prepandemic samples.
