# Supplementary material for: Antibody Binding and Neutralization of Live SARS-CoV-2 Variants Including BA.4/5 Following Booster Vaccination of Patients with B-cell Malignancies
Source: Cancer Res Commun. 2022 Dec 22;2(12):1684–92. doi: 10.1158/2767-9764.CRC-22-0471 (PMC9833496; doi:10.1158/2767-9764.CRC-22-0471)
Supplement: Supplementary Figure SF4 — Supplemental Figure 4. Anti-spike binding IgG titers after booster correlate with conventional B cell numbers in the blood prior booster. [file crc-22-0471-s07.pdf]

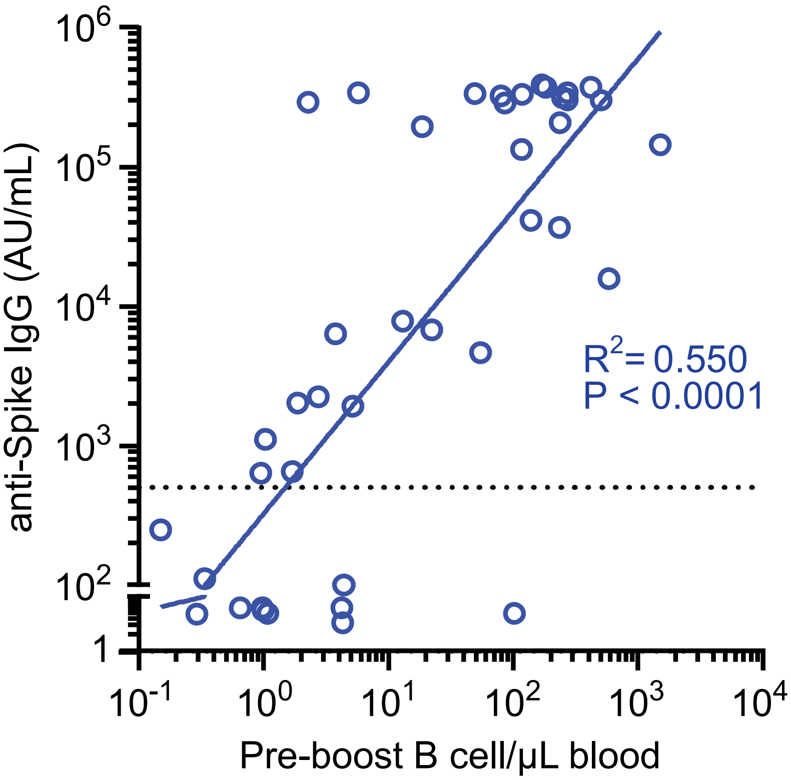

Supplemental Figure 4. Anti-spike binding IgG titers after booster correlate with conventional B cell numbers in the blood prior booster. Conventional B cells are defined as CD19+CD20+CD5-CD3- lymphocytes and detected by flow cytometry. Horizontal dotted line = background antibody levels determined from pre-pandemic samples. Correlation was statistically significant using extra-sum-of-squares F test.
