# Supplementary material for: Antibody Binding and Neutralization of Live SARS-CoV-2 Variants Including BA.4/5 Following Booster Vaccination of Patients with B-cell Malignancies
Source: Cancer Res Commun. 2022 Dec 22;2(12):1684–92. doi: 10.1158/2767-9764.CRC-22-0471 (PMC9833496; doi:10.1158/2767-9764.CRC-22-0471)
Supplement: Supplementary Figure SF5 — Supplemental Figure 5. Reduced IgG binding titers against the spike protein of SARS-CoV-2 variants in NHL/CLL patients. [file crc-22-0471-s08.pdf]

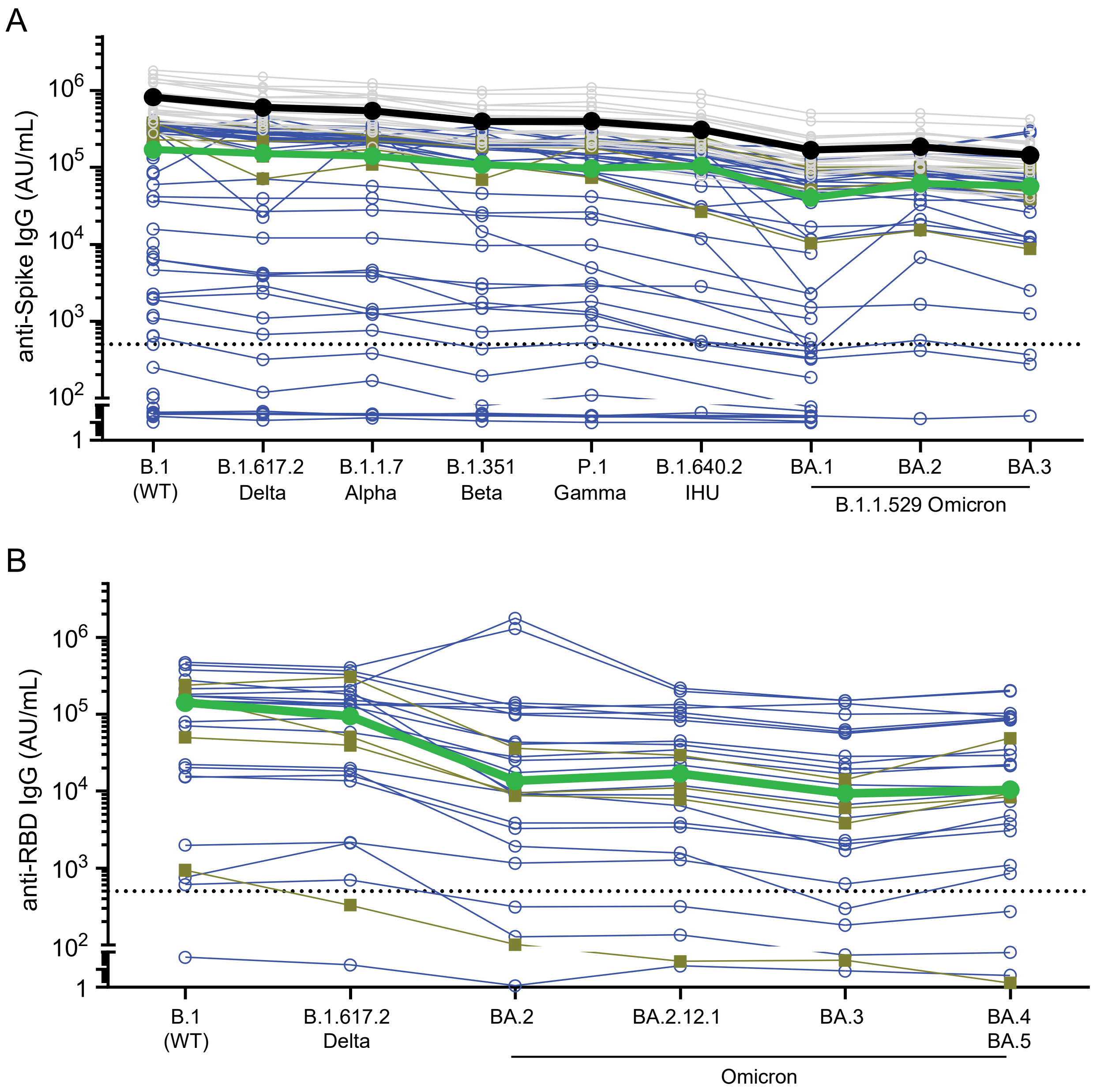

Supplemental Figure 5. Reduced IgG binding titers against the spike protein of SARS-CoV-2 variants in NHL/CLL patients. A) Anti-spike binding titers against SARS-CoV-2 variants among healthy (gray), nucleocapsid-negative (blue), and nucleocapsid-positive (gold) NHL/CLL patients. Median titers in healthy (black) and NHL/CLL patients (green) are also shown. Titers against Omicron variants are significantly reduced compared to WT and earlier variants but are higher in healthy controls than in NHL/CLL patients. B) Anti-spike RBD binding titers against SARS-CoV-2 Omicron variants are lower when compared to RBD from WT. Horizontal dotted line = background antibody levels determined from pre-pandemic samples.
